# Area Level Deprivation Is an Independent Determinant of Prevalent Type 2 Diabetes and Obesity at the National Level in Germany. Results from the National Telephone Health Interview Surveys ‘German Health Update’ GEDA 2009 and 2010

**DOI:** 10.1371/journal.pone.0089661

**Published:** 2014-02-27

**Authors:** Werner Maier, Christa Scheidt-Nave, Rolf Holle, Lars E. Kroll, Thomas Lampert, Yong Du, Christin Heidemann, Andreas Mielck

**Affiliations:** 1 Helmholtz Zentrum München, German Research Center for Environmental Health (GmbH), Institute of Health Economics and Health Care Management, Neuherberg, Germany; 2 Robert Koch Institute, Berlin, Germany; Iran University of Medical Sciences, Iran (Islamic Republic of)

## Abstract

**Objective:**

There is increasing evidence that prevention programmes for type 2 diabetes mellitus (T2DM) and obesity need to consider individual and regional risk factors. Our objective is to assess the independent association of area level deprivation with T2DM and obesity controlling for individual risk factors in a large study covering the whole of Germany.

**Methods:**

We combined data from two consecutive waves of the national health interview survey ‘GEDA’ conducted by the Robert Koch Institute in 2009 and 2010. Data collection was based on computer-assisted telephone interviews. After exclusion of participants <30 years of age and those with missing responses, we included n = 33,690 participants in our analyses. The outcome variables were the 12-month prevalence of known T2DM and the prevalence of obesity (BMI ≥30 kg/m^2^). We also controlled for age, sex, BMI, smoking, sport, living with a partner and education. Area level deprivation of the districts was defined by the German Index of Multiple Deprivation. Logistic multilevel regression models were performed using the software SAS 9.2.

**Results:**

Of all men and women living in the most deprived areas, 8.6% had T2DM and 16.9% were obese (least deprived areas: 5.8% for T2DM and 13.7% for obesity). For women, higher area level deprivation and lower educational level were both independently associated with higher T2DM and obesity prevalence [highest area level deprivation: OR 1.28 (95% CI: 1.05–1.55) for T2DM and OR 1.28 (95% CI: 1.10–1.49) for obesity]. For men, a similar association was only found for obesity [OR 1.20 (95% CI: 1.02–1.41)], but not for T2DM.

**Conclusion:**

Area level deprivation is an independent, important determinant of T2DM and obesity prevalence in Germany. Identifying and targeting specific area-based risk factors should be considered an essential public health issue relevant to increasing the effectiveness of diabetes and obesity prevention.

## Introduction

There is sound evidence that the prevalence of type 2 diabetes mellitus (T2DM) is strongly associated with obesity and that both T2DM and obesity are inversely associated with individual socio-economic status (SES) [1]–[4]. Also, results from recent studies indicate that higher area level deprivation is significantly associated with greater risk of abnormal glucose tolerance and higher prevalence of T2DM and obesity, independently of individual SES [5], [6], and that moving from a more deprived to a less deprived neighbourhood is associated with reductions in the prevalence of diabetes and obesity [7]. In order to reduce the burden of T2DM, obesity and associated health care costs [8], it would therefore be plausible to target individual as well as area-based risk factors.

In Germany, as in all industrialized countries, T2DM poses a major public health problem. According to the International Diabetes Federation (IDF), the prevalence of diagnosed diabetes in Germany is still lower than in the US, but higher than in a number of other European countries [9], [10]. Regional differences in the prevalence of T2DM within Germany have been reported previously in a study using data from the research consortium DIAB-CORE (Diabetes-Collaborative Research of Epidemiologic Studies) cooperating in the Competence Network for Diabetes mellitus (‘Kompetenznetz Diabetes mellitus’), and these differences seem to be higher in the east than in the west [11]. Two other studies based on this dataset indicate that regional differences in T2DM prevalence can partially be explained by the degree of area level deprivation at a municipality level [6] and by the neighbourhood unemployment rate in cities [12], independently of individual SES and established diabetes risk factors. Sex-specific differences in the associations with T2DM have been reported as well [6], [13]. However, these results were based on five regional surveys conducted in different parts of Germany at different points in time. At the national level, variation in the prevalence of T2DM and obesity has been demonstrated for German districts using small area estimation techniques [14]. However, this previous study did not consider individual characteristics, except age, sex and household size.

Studies from other countries are often limited as well, because they did not adjust for individual SES [15], [16], used only a proxy for individual SES or did not adjust for body mass index (BMI) [17].

Against this background, the present study aimed to (1) determine whether area level deprivation is associated with the prevalence of T2DM and obesity in Germany at the national level, independently of individual risk factors and (2) further explore sex-specific differences.

## Methods

### Study population

The German Health Update (‘Gesundheit in Deutschland Aktuell’, GEDA) survey system consists of periodically repeated representative national health interview surveys. GEDA is an integral part of the continuous health monitoring conducted by the Robert Koch Institute. We used cross-sectional data from the 2009 and 2010 GEDA survey waves, which were conducted between July 2008 and June 2009 (GEDA 2009) and between September 2009 and July 2010 (GEDA 2010) using highly standardized computer-assisted telephone interview (CATI) techniques. The methods have been described in detail previously [18]–[20]. In brief, the target population comprised adults aged 18 years and older living in private households with a landline, who were able to understand and speak German. Random sampling at the household level is based on the Gabler–Häder method applying random digit dialling [21], and random selection of individuals was achieved by the last birthday procedure [22]. According to the standard definitions of the American Association for Public Opinion Research (AAPOR) [23], the calculated cooperation rates at respondent level were 51.2% for GEDA 2009 and 55.8% for GEDA 2010, and the response rates 3 (AAPOR) quoting the number of realized interviews proportional to all possible households were 29.1% for GEDA 2009 and 28.9% for GEDA 2010 [24], [25]. We pooled the n = 21,262 participants (women n = 12,114) in GEDA 2009 and n = 22,050 participants in GEDA 2010 (women n = 12,483), which resulted in a combined dataset of n = 43,312 participants (women n = 24,597).

We intended to target T2DM but the assessment of diabetes in the GEDA surveys allows no distinction between different types of diabetes. T2DM accounts for about 90–95% of all diabetes cases [26] and is relatively rare in younger age groups. For the present analysis, we therefore excluded n = 7,620 participants below the age of 30 years. In our sample, the diabetes prevalence in the age group 18–29 years was less than 1% (n = 62, 0.8%). We also excluded n = 2,002 participants because of missing information on diabetes, BMI, the district of residence, educational level, lifestyle (smoking status, sport activity) and living with a partner. Thus, the final study included n = 33,690 people (14,402 men and 19,288 women).

### Ethics Statement

As the participation in the National Health Telephone Interview Surveys is voluntary, not arising any costs for survey participants, and because the study has no medical relevance for individual survey participants (no medical research involving human subjects is being conducted) an ethics approval was not compulsory. In terms of data protection and informed consent the study was approved by The Federal Commissioner for Data Protection and Freedom of Information. Verbal informed consent was provided by all participants prior to the interview.

### Individual level variables

Information regarding individual data is based on self-report as collected by CATI. For the present analysis, we included information on sex and chronological age, history of known diabetes mellitus, BMI, smoking status, sport activity, living with a partner and educational level.

Study participants were asked whether they had ever been diagnosed with diabetes by a physician (lifetime diagnosis) and, if yes, whether they had also been suffering from diabetes in the past 12 months. We defined the dependent variable ‘12-month prevalence of diabetes’ as a positive answer to both questions, which we used in order to reduce misclassification bias due to under- or overreporting. T2DM was defined by restricting the dataset as described above. Obesity was defined as a BMI ≥30 kg/m^2^, based on self-reported weight and height [27].

In order to control for potential confounding, the following covariates were included in our analysis: sex, age (three categories: 30–49, 50–64 and ≥65 years), BMI (three categories: <25 kg/m^2^, 25 to <30 kg/m^2^ and ≥30 kg/m^2^), sport activity (measured by hours of sport activity, three categories: no sport activity, up to 4 hours/week, more than 4 hours/week) [28] and smoking status (three categories: never smoker, ex-smoker and current smoker) [29]. Potential social support from a partner could have strong associations with the prevalence of T2DM [30], so we also adjusted for living with a partner (two categories: living with a partner, living without a partner).

Individual SES was defined by educational level [3], [4]. The participants were asked for their highest level of school qualification obtained. We classified educational level as a variable with three categories contrasting low, medium and high levels. According to the German school system, low educational level includes participants with up to 9 years of schooling or having left school without having obtained any formal qualifications. Medium educational level is equivalent to 10 years of schooling and high educational level to 12 or 13 years of schooling, which is required to enter university.

### Area level deprivation

Area level deprivation was assessed by the German Index of Multiple Deprivation (GIMD), a recently introduced area-based deprivation measure that has not yet been applied to the GEDA dataset. The GIMD has been established based on the method used in the UK [31] and adapted to the German context [32], including seven domains of deprivation (i.e. income, employment, education, municipal or district revenue, social capital, environment, security). The GIMD already showed significant associations with T2DM, reported in a previous study confined to five regional surveys [6], and with other health-related outcomes [33], [34]. For the present study, we calculated the GIMD scores for all urban and rural districts covering the whole of Germany. The population size of urban and rural districts ranges from about 35,000 up to more than one million inhabitants (e.g. cities such as Hamburg or Berlin). We assigned the districts to deprivation quintiles, with quintile 1 including the least deprived and quintile 5 the most deprived districts. Information on the deprivation status of the district of residence was assigned to each study participant. Overall, 412 districts were included in our analyses. For reasons of data protection, the analyses are based on anonymized individual level data and districts were pseudonymized, excluding identification and localization of the respective districts.

### Statistical analysis

We carried out univariate and bivariate analyses calculating chi-square statistics and Cochran–Armitage tests for trend. Then we performed logistic multilevel regression models and fitted two-level binomial logit-link models (level 1: individuals; level 2: districts) with random intercept, calculating first crude odds ratios (ORs) with their 95% confidence intervals (95% CIs). We tested for associations between district deprivation and the prevalence of T2DM (12-month prevalence) and obesity in subsequent models, controlling for potential confounding or effect modification. Sex-specific results were obtained by stratified analysis. Finally, we report ORs with their 95% CIs and area level variances (V_A_) with their standard errors (SE). In order to quantify the relevance of area level variation, we also calculated the median odds ratios (MORs), which can be calculated as a simple function of the area level variance V_A_ [35]: MOR  =  exp [√(2 × V_A_) × 0.6745].

All analyses were performed as complete case analysis using the software SAS 9.2 (SAS Institute Inc., Cary, NC, USA). The logistic multilevel models were estimated with the SAS procedure GLIMMIX, using a maximum likelihood estimation based on Laplace approximation.

## Results

Table 1 shows the distribution of the individual level and area level characteristics in our combined final sample of n = 33,690 participants (women n = 19,288). Table 2 presents the prevalence of T2DM and obesity by individual and area level characteristics. The participants were distributed across all districts of Germany (n = 412). The most deprived districts were mainly found in the east of Germany, but also in some western areas (e.g. the Ruhr area), whereas the least deprived districts were mainly found in the south and south-west, but also in the north-west of the country (data not shown). The overall 12-month prevalence (referred to as prevalence in the following) of T2DM and the prevalence of obesity remained virtually unchanged between 2009 and 2010 (T2DM: 6.8% in 2009 and 6.7% in 2010; obesity: 15.0% in 2009 and 15.4% in 2010). Chi-square tests mainly showed highly significant associations between the dependent variables (T2DM and obesity) and all independent variables (see Table 2). Also, there is a stepwise increase in the prevalence of T2DM and obesity with increasing area deprivation for women. Cochran–Armitage tests displayed highly significant trends (p<0.0001, two-sided) between T2DM and obesity, on the one hand, and educational level or area level deprivation, on the other.

**Table 1:** Distribution of individual level and area level characteristics in the pooled GEDA sample (2009 and 2010).

	Men	Women	Total
Participants (n)	14,402	19,288	33,690
T2DM^a^ (%)	7.7	6.1	6.8
Obesity^b^ (%)	16.4	14.2	15.2
Independent variables (%)			
Age (years)			
30–49	46.8	47.7	47.3
50–64	29.6	30.2	29.9
≥65	23.6	22.2	22.8
BMI (kg/m^2^)			
<25	36.6	56.3	47.9
25–<30	47.0	29.5	37.0
≥30	16.4	14.2	15.2
Smoking status			
never smoker	35.6	50.2	44.0
ex-smoker	34.8	24.9	29.1
current smoker	29.6	24.9	26.9
Sport activity			
>4 h/week	24.4	19.2	21.4
up to 4 h/week	41.2	49.5	46.0
no sport activity	34.4	31.3	32.6
Partner			
living with a partner	74.3	65.9	69.5
living without a partner	25.7	34.1	30.5
Educational level			
high level	46.9	38.4	42.0
medium level	25.1	34.6	30.5
low level	28.0	27.1	27.5
GIMD quintiles (Q)			
Q1 ( = least deprived)	24.0	23.6	23.8
Q2	21.9	21.6	21.8
Q3	19.4	19.3	19.4
Q4	16.6	17.3	17.0
Q5 ( = most deprived)	18.1	18.2	18.1

a Crude 12-month prevalence of type 2 diabetes; ^b^ crude prevalence of obesity (BMI ≥30 kg/m^2^).

**Table 2:** Prevalence of type 2 diabetes and obesity by individual level and area level characteristics in the pooled GEDA sample (2009 and 2010).

	T2DM^a^			Obesity^b^		
	Men	Women	Total	Men	Women	Total
Participants (n)	14,402	19,288	33,690	14,402	19,288	33,690
Independent variables (%)						
Age (years)						
30–49	1.8	1.7	1.8	13.5	10.5	11.8
50–64	9.6	6.7	7.9	20.8	17.4	18.8
≥65	17.2	14.6	15.8	16.8	18.0	17.5
	P<0.0001	P<0.0001	P<0.0001	P<0.0001	P<0.0001	P<0.0001
BMI (kg/m^2^)						
<25	3.3	2.3	2.7	–	–	–
25–<30	7.3	6.9	7.2	–	–	–
≥30	18.7	19.1	18.9	–	–	–
	P<0.0001	P<0.0001	P<0.0001			
Smoking status						
never smoker	6.2	6.9	6.6	13.8	14.9	14.5
ex-smoker	11.7	6.4	9.1	21.0	16.0	18.6
current smoker	4.9	4.1	4.5	14.1	11.2	12.6
	P<0.0001	P<0.0001	P<0.0001	P<0.0001	P<0.0001	P<0.0001
Sport activity						
>4 h/week	6.1	4.2	5.1	11.2	9.3	10.2
up to 4 h/week	5.6	4.0	4.7	14.1	12.1	12.9
no sport activity	11.5	10.4	10.9	22.8	20.7	21.7
	P<0.0001	P<0.0001	P<0.0001	P<0.0001	P<0.0001	P<0.0001
Partner						
living with a partner	7.5	4.6	5.9	16.4	13.2	14.7
living without a partner	8.4	9.0	8.8	16.4	16.3	16.3
	P = 0.109	P<0.0001	P<0.0001	P = 0.969	P<0.0001	P<0.0001
Educational level						
high level	5.6	2.9	4.2	11.8	8.6	10.1
medium level	6.7	4.4	5.2	17.6	13.4	14.9
low level	12.3	12.6	12.5	23.1	23.4	23.3
	P<0.0001	P<0.0001	P<0.0001	P<0.0001	P<0.0001	P<0.0001
GIMD quintiles (Q)						
Q1 ( = least deprived)	6.9	4.9	5.8	14.9	12.7	13.7
Q2	6.9	5.4	6.0	15.5	13.5	14.4
Q3	8.2	5.8	6.8	17.9	14.1	15.7
Q4	8.1	6.5	7.2	16.8	15.2	15.9
Q5 ( = most deprived)	9.1	8.3	8.6	17.7	16.4	16.9
	P = 0.008	P<0.0001	P<0.0001	P = 0.004	P<0.0001	P<0.0001

a Crude 12-month prevalence of type 2 diabetes; ^b^ crude prevalence of obesity (BMI ≥30 kg/m^2^).

P values: Chi-square test.

Models for both men and women combined (Tables 3 and 4) showed significant associations between the prevalence of T2DM and obesity, on the one hand, and area level deprivation, on the other, independent of individual educational level. Comparing the most deprived quintile 5 with the least deprived quintile 1, estimates remained statistically significant for T2DM [OR 1.18 (95% CI: 1.03–1.35)] and for obesity [OR 1.27 (95% CI: 1.12–1.42)] in the fully adjusted models. It is important to point out that most ORs clearly increased with increasing area level deprivation. Additional modelling with age as a continuous variable showed almost identical results for area level deprivation. Tests for linear trend in the full models for men and women combined showed significant P values (T2DM: P = 0.0094; obesity: P<0.0001). Low educational level was significantly associated with increased prevalence of T2DM, and the same applies to overweight (BMI ≥25 and <30 kg/m^2^) and obesity (BMI ≥30 kg/m^2^).

**Table 3:** Associations between type 2 diabetes, area level deprivation and individual level variables for men and women (n = 33,690).

	Men and women			
Variables	Crude	Model 1	Model 2	Model 3
	OR (95% CI)	OR (95% CI)	OR (95% CI)	OR (95% CI)
Sex (*women)				
men	1.30 (1.20–1.42)	1.29 (1.18–1.40)	1.32 (1.20–1.45)	1.19 (1.08–1.31)
Age (*30–49 years)				
50–64	4.80 (4.18–5.52)	4.78 (4.16–5.49)	4.06 (3.52–4.67)	3.67 (3.18–4.23)
≥65	10.48 (9.17–11.98)	10.31 (9.02–11.79)	7.16 (6.21–8.25)	7.30 (6.32–8.43)
BMI (*<25 kg/m^2^)				
25–<30	2.81 (2.50–3.16)	–	–	2.09 (1.85–2.36)
≥30	8.54 (7.58–9.61)	–	–	5.96 (5.26–6.76)
Smoking status (*never smoker)				
ex-smoker	1.42 (1.29–1.56)	–	1.31 (1.18–1.45)	1.24 (1.12–1.38)
current smoker	0.66 (0.59–0.75)	–	0.81 (0.72–0.93)	0.96 (0.84–1.09)
Sport activity (*>4 h/week)				
up to 4 h/week	0.90 (0.79–1.03)	–	1.04 (0.91–1.18)	0.93 (0.81–1.06)
no sport activity	2.26 (2.00–2.55)	–	1.92 (1.70–2.18)	1.52 (1.34–1.73)
Partner (*living with a partner)				
living without a partner	1.53 (1.40–1.67)	–	1.29 (1.17–1.41)	1.30 (1.18–1.43)
Educational level (*high level)				
medium level	1.25 (1.10–1.40)	–	1.25 (1.11–1.42)	1.14 (1.00–1.29)
low level	3.29 (2.96–3.64)	–	1.89 (1.70–2.11)	1.49 (1.33–1.67)
GIMD quintiles (*Q1 = least deprived)				
Q2	1.05 (0.91–1.21)	1.05 (0.91–1.21)	1.00 (0.87–1.15)	0.99 (0.86–1.15)
Q3	1.18 (1.02–1.37)	1.19 (1.03–1.37)	1.11 (0.97–1.28)	1.08 (0.94–1.25)
Q4	1.27 (1.10–1.48)	1.21 (1.04–1.40)	1.14 (0.99–1.32)	1.08 (0.93–1.25)
Q5 ( = most deprived)	1.59 (1.37–1.84)	1.37 (1.19–1.58)	1.26 (1.10–1.45)	1.18 (1.03–1.35)
Variances				
V_A_ (SE)	–	0.011 (0.011)	0	0
MOR	–	1.11	1.00	1.00

*: Reference group; OR (odds ratios); 95% CI (95% confidence intervals); bold type = significant.

V_A_ (area level variance); SE (standard error); MOR (median odds ratio).

**Model 0:** crude OR (95% CI), unadjusted odds ratios with their 95% confidence intervals

**Model 1**: area level deprivation, adjusted for sex and age

**Model 2**: additionally adjusted for educational level, lifestyle covariates (excl. BMI) and partner

**Model 3**: additionally adjusted for BMI

**Table 4:** Associations between obesity, area level deprivation and individual level variables for men and women (n = 33,690).

	Men and women		
Variables	Crude	Model 1	Model 2
	OR (95% CI)	OR (95% CI)	OR (95% CI)
Sex (*women)			
men	1.18 (1.12–1.26)	1.18 (1.11–1.26)	1.22 (1.14–1.30)
Age (*30–49 years)			
50–64	1.74 (1.62–1.86)	1.73 (1.61–1.86)	1.44 (1.34–1.55)
≥65	1.60 (1.48–1.72)	1.58 (1.46–1.70)	1.03 (0.95–1.12)
Smoking status (*never smoker)			
ex-smoker	1.35 (1.26–1.45)	–	1.27 (1.18–1.36)
current smoker	0.85 (0.78–0.92)	–	0.71 (0.65–0.77)
Sport activity (*>4 h/week)			
up to 4 h/week	1.30 (1.18–1.42)	–	1.37 (1.25–1.50)
no sport activity	2.42 (2.21–2.64)	–	2.24 (2.05–2.46)
Partner (*living with a partner)			
living without a partner	1.16 (1.08–1.23)	–	1.15 (1.07–1.23)
Educational level (*high level)			
medium level	1.54 (1.42–1.66)	–	1.53 (1.41–1.66)
low level	2.67 (2.48–2.88)	–	2.33 (2.16–2.53)
GIMD quintiles (*Q1 = least deprived)			
Q2	1.07 (0.95–1.20)	1.06 (0.94–1.19)	1.03 (0.92–1.15)
Q3	1.19 (1.06–1.34)	1.19 (1.06–1.34)	1.14 (1.02–1.27)
Q4	1.21 (1.07–1.37)	1.20 (1.06–1.35)	1.17 (1.04–1.32)
Q5 ( = most deprived)	1.38 (1.23–1.56)	1.33 (1.18–1.50)	1.27 (1.12–1.42)
Variances			
V_A_ (SE)	–	0.043 (0.010)	0.031 (0.009)
MOR	–	1.22	1.18

*: Reference group; OR (odds ratios); 95% CI (95% confidence intervals); bold type = significant.

V_A_ (area level variance); SE (standard error); MOR (median odds ratio).

**Model 0:** crude OR (95% CI), unadjusted odds ratios with their 95% confidence intervals

**Model 1**: area level deprivation, adjusted for sex and age

**Model 2**: additionally adjusted for educational level, lifestyle covariates and partner

Separate analyses revealed important differences between men and women for the prevalence of T2DM (Tables 5 and 6). A significant association with area level deprivation could be seen only for women: in the most deprived districts, their risk of having T2DM was clearly higher compared with those in the least deprived districts, even after controlling for individual educational level [OR 1.28 (95% CI: 1.05–1.55)]. Educational level showed a clear stepwise increase in the risk of having T2DM, but was about 2.5-fold higher for female participants with low educational level than for male participants (OR 1.77 vs. OR 1.31).

**Table 5:** Associations between type 2 diabetes, area level deprivation and individual level variables for men (n = 14,402).

	Men			
Variables	Crude	Model 1	Model 2	Model 3
	OR (95% CI)	OR (95% CI)	OR (95% CI)	OR (95% CI)
Age (*30–49 years)				
50–64	5.77 (4.69–7.10)	5.77 (4.69–7.09)	5.02 (4.07–6.19)	4.65 (3.77–5.75)
≥65	11.39 (9.32–13.92)	11.34 (9.28–13.86)	8.87 (7.19–10.95)	9.42 (7.61–11.66)
BMI (*<25 kg/m^2^)				
25–<30	2.29 (1.92–2.74)	–	–	1.90 (1.59–2.28)
≥30	6.65 (5.54–7.98)	–	–	5.19 (4.28–6.30)
Smoking status (*never smoker)				
ex-smoker	1.99 (1.73–2.29)	–	1.48 (1.28–1.72)	1.35 (1.16–1.57)
current smoker	0.78 (0.65–0.93)	–	0.89 (0.74–1.08)	0.98 (0.81–1.19)
Sport activity (*>4 h/week)				
up to 4 h/week	0.93 (0.78–1.10)	–	1.06 (0.89–1.27)	0.98 (0.81–1.17)
no sport activity	2.00 (1.70–2.36)	–	1.71 (1.44–2.03)	1.42 (1.19–1.69)
Partner (*living with a partner)				
living without a partner	1.12 (0.98–1.28)	–	1.24 (1.08–1.44)	1.28 (1.11–1.49)
Educational level (*high level)				
medium level	1.22 (1.03–1.44)	–	1.33 (1.12–1.58)	1.23 (1.03–1.46)
low level	2.40 (2.08–2.76)	–	1.53 (1.32–1.77)	1.31 (1.12–1.52)
GIMD quintiles (*Q1 = least deprived)				
Q2	1.00 (0.82–1.20)	0.98 (0.80–1.19)	0.93 (0.77–1.14)	0.92 (0.76–1.13)
Q3	1.20 (0.99–1.45)	1.21 (1.00–1.47)	1.13 (0.93–1.38)	1.07 (0.88–1.31)
Q4	1.18 (0.97–1.43)	1.16 (0.95–1.42)	1.10 (0.90–1.36)	1.04 (0.85–1.29)
Q5 ( = most deprived)	1.34 (1.11–1.61)	1.19 (0.98–1.44)	1.12 (0.92–1.36)	1.07 (0.88–1.30)
Variances				
V_A_ (SE)	–	0	0	0
MOR	–	1.00	1.00	1.00

*: Reference group; OR (odds ratios); 95% CI (95% confidence intervals); bold type = significant.

V_A_ (area level variance); SE (standard error); MOR (median odds ratio).

**Model 0:** crude OR (95% CI), unadjusted odds ratios with their 95% confidence intervals

**Model 1**: area level deprivation, adjusted for age

**Model 2**: additionally adjusted for educational level, lifestyle covariates (excl. BMI) and partner

**Model 3**: additionally adjusted for BMI

**Table 6:** Associations between type 2 diabetes, area level deprivation and individual level variables for women (n = 19,288).

	Women			
Variables	Crude	Model 1	Model 2	Model 3
	OR (95% CI)	OR (95% CI)	OR (95% CI)	OR (95% CI)
Age (*30–49 years)				
50–64	4.09 (3.39–4.94)	4.05 (3.36–4.89)	3.27 (2.69–3.96)	2.88 (2.37–3.50)
≥65	9.76 (8.16–11.67)	9.53 (7.96–11.40)	5.50 (4.51–6.71)	5.45 (4.45–6.67)
BMI (*<25 kg/m^2^)				
25–<30	3.10 (2.64–3.65)	–	–	2.19 (1.85–2.58)
≥30	9.89 (8.45–11.58)	–	–	6.44 (5.45–7.61)
Smoking status (*never smoker)				
ex-smoker	0.95 (0.82–1.09)	–	1.18 (1.02–1.37)	1.14 (0.98–1.33)
current smoker	0.59 (0.50–0.69)	–	0.77 (0.65–0.92)	0.95 (0.80–1.14)
Sport activity (*>4 h/week)				
up to 4 h/week	0.96 (0.79–1.16)	–	1.04 (0.86–1.26)	0.91 (0.75–1.11)
no sport activity	2.64 (2.20–3.16)	–	2.17 (1.80–2.61)	1.64 (1.36–1.99)
Partner (*living with a partner)				
living without a partner	2.07 (1.84–2.34)	–	1.33 (1.17–1.52)	1.34 (1.17–1.53)
Educational level (*high level)				
medium level	1.51 (1.26–1.81)	–	1.31 (1.09–1.57)	1.14 (0.95–1.38)
low level	4.80 (4.09–5.62)	–	2.44 (2.06–2.89)	1.77 (1.49–2.10)
GIMD quintiles (*Q1 = least deprived)				
Q2	1.10 (0.90–1.35)	1.12 (0.91–1.37)	1.09 (0.89–1.33)	1.08 (0.88–1.32)
Q3	1.19 (0.97–1.45)	1.17 (0.95–1.44)	1.10 (0.90–1.35)	1.11 (0.90–1.36)
Q4	1.37 (1.12–1.68)	1.26 (1.03–1.55)	1.17 (0.96–1.43)	1.12 (0.91–1.38)
Q5 ( = most deprived)	1.82 (1.49–2.21)	1.54 (1.27–1.89)	1.39 (1.14–1.69)	1.28 (1.05–1.55)
Variances				
V_A_ (SE)	–	0.022 (0.021)	0.004 (0.021)	0
MOR	–	1.15	1.06	1.00

*: Reference group; OR (odds ratios); 95% CI (95% confidence intervals); bold type = significant.

V_A_ (area level variance); SE (standard error); MOR (median odds ratio).

**Model 0**: crude OR (95% CI), unadjusted odds ratios with their 95% confidence intervals

**Model 1**: area level deprivation, adjusted for age

**Model 2**: additionally adjusted for educational level, lifestyle covariates (excl. BMI) and partner

**Model 3**: additionally adjusted for BMI

In contrast, the stratified analyses for obesity (Tables 7 and 8) showed similar results for both men and women in the most deprived districts: the risk of being obese was clearly higher than in the least deprived districts, even after controlling for individual educational level [OR 1.20 (95% CI: 1.02–1.41) for men and OR 1.28 (95% CI: 1.10–1.49) for women].

**Table 7:** Associations between obesity, area level deprivation and individual level variables for men (n = 14,402).

	Men		
Variables	Crude	Model 1	Model 2
	OR (95% CI)	OR (95% CI)	OR (95% CI)
Age (*30–49 years)			
50–64	1.68 (1.52–1.87)	1.68 (1.52–1.86)	1.41 (1.27–1.57)
≥65	1.31 (1.16–1.46)	1.30 (1.16–1.46)	0.93 (0.82–1.06)
Smoking status (*never smoker)			
ex-smoker	1.66 (1.50–1.84)	–	1.49 (1.34–1.66)
current smoker	1.02 (0.91–1.15)	–	0.84 (0.74–0.95)
Sport activity (*>4 h/week)			
up to 4 h/week	1.30 (1.14–1.48)	–	1.33 (1.17–1.51)
no sport activity	2.33 (2.06–2.64)	–	2.14 (1.88–2.43)
Partner (*living with a partner)			
living without a partner	1.02 (0.92–1.13)	–	1.06 (0.95–1.18)
Educational level (*high level)			
medium level	1.57 (1.40–1.76)	–	1.51 (1.34–1.70)
low level	2.22 (2.00–2.46)	–	1.90 (1.70–2.12)
GIMD quintiles (*Q1 = least deprived)			
Q2	1.05 (0.90–1.23)	1.04 (0.89–1.22)	0.99 (0.86–1.15)
Q3	1.24 (1.06–1.46)	1.25 (1.06–1.46)	1.18 (1.01–1.37)
Q4	1.15 (0.98–1.36)	1.15 (0.97–1.36)	1.12 (0.96–1.32)
Q5 ( = most deprived)	1.31 (1.11–1.55)	1.28 (1.08–1.51)	1.20 (1.02–1.41)
Variances			
V_A_ (SE)	–	0.049 (0.017)	0.025 (0.015)
MOR	–	1.24	1.16

*: Reference group; OR (odds ratios); 95% CI (95% confidence intervals); bold type = significant.

V_A_ (area level variance); SE (standard error); MOR (median odds ratio).

**Model 0**: crude OR (95% CI), unadjusted odds ratios with their 95% confidence intervals

**Model 1**: area level deprivation, adjusted for age

**Model 2**: additionally adjusted for educational level, lifestyle covariates and partner

**Table 8:** Associations between obesity, area level deprivation and individual level variables for women (n = 19,288).

	Women		
Variables	Crude	Model 1	Model 2
	OR (95% CI)	OR (95% CI)	OR (95% CI)
**Age** (*30–49 years)			
50–64	**1.79** (1.63–1.97)	**1.78** (1.62–1.96)	**1.43** (1.30–1.59)
≥65	**1.87** (1.69–2.08)	**1.85** (1.67–2.05)	1.05 (0.93–1.18)
**Smoking status** (*never smoker)			
ex-smoker	**1.10** (1.00–1.21)	–	**1.16** (1.05–1.28)
current smoker	**0.72** (0.65–0.81)	–	**0.64** (0.57–0.72)
**Sport activity** (*>4 h/week)			
up to 4 h/week	**1.34** (1.18–1.52)	–	**1.41** (1.24–1.60)
no sport activity	**2.55** (2.25–2.90)	–	**2.37** (2.08–2.70)
**Partner** (*living with a partner)			
living without a partner	**1.30** (1.19–1.41)	–	**1.18** (1.08–1.29)
**Educational level** (*high level)			
medium level	**1.65** (1.48–1.84)	–	**1.61** (1.44–1.80)
low level	**3.26** (2.93–3.62)	–	**2.82** (2.52–3.16)
**GIMD quintiles** (*Q1 = least deprived)			
Q2	1.08 (0.93–1.25)	1.08 (0.93–1.25)	1.06 (0.92–1.22)
Q3	1.13 (0.98–1.32)	1.13 (0.97–1.31)	1.09 (0.94–1.27)
Q4	**1.25** (1.08–1.46)	**1.22** (1.05–1.42)	**1.20** (1.03–1.39)
Q5 ( = most deprived)	**1.43** (1.22–1.66)	**1.35** (1.16–1.57)	**1.28** (1.10–1.49)
**Variances**			
V_A_ (SE)	–	0.045 (0.015)	0.034 (0.015)
MOR	–	1.22	1.19

*: Reference group; OR (odds ratios); 95% CI (95% confidence intervals); bold type = significant.

V_A_ (area level variance); SE (standard error); MOR (median odds ratio).

**Model 0**: crude OR (95% CI), unadjusted odds ratios with their 95% confidence intervals

**Model 1**: area level deprivation, adjusted for age

**Model 2**: additionally adjusted for educational level, lifestyle covariates and partner

Differences between area variances (V_A_, MOR) were generally low in the T2DM models. In the obesity models, there was a larger variation between districts, but this was quite similar for men and women (Tables 3 to 8).

## Discussion

Our objective was to evaluate the relationship between area level deprivation and the prevalence of T2DM and obesity, looking also at the role of educational level and potential differences between men and women. Our findings suggest that living in very deprived districts and having a low educational level are both independently associated with a higher prevalence of T2DM and a higher prevalence of obesity. The increased prevalence of obesity in these highly deprived areas applies to both men and women, but the increased prevalence of T2DM in the most deprived districts is confined to women. Also, the increased prevalence of T2DM and obesity associated with low educational level is stronger for women than for men. Concerning models with the dependent variable T2DM, it is important to note that controlling for BMI (like other covariates, e.g. smoking) may lead to potential overadjustment, and BMI could act as an important intermediate factor between area level deprivation and T2DM [36]. Models not including BMI should therefore be considered as well. Concerning the higher prevalence of T2DM and obesity for ex-smokers compared with current smokers, it should be noted that smoking cessation can result in weight gain and lead to higher diabetes risk [37]. Also, chronic conditions may generally lead to enforced smoking cessation and heavy smoking to premature mortality.

Our findings are in good agreement with results reported from other countries. In the Diabetes Study of Northern California (DISTANCE), Laraia et al. [38] found that higher levels of neighbourhood deprivation were positively associated with cardiometabolic risk factors such as BMI, suggesting an association between deprivation and individual health outcomes. Based on data from Scotland, Wild and colleagues [39] stated that the burden of diabetes and the prevalence of obesity were higher in more deprived populations and that deprivation was associated with failure to reach cholesterol targets in secondary prevention. In the Australian Diabetes, Obesity and Lifestyle (AusDiab) study, Williams et al. [5] showed that area deprivation predicted the development of abnormal glucose metabolism (AGM). Bocquier et al. [17] reported a positive association between area deprivation and treated diabetes in south-eastern France, drawing attention to the localization of priority areas for diabetes prevention and the necessity of explaining the mechanisms behind the association between area level deprivation and diabetes. Only a few of these results were adjusted for individual SES, such as education [5], [40].

In our analyses, controlling for individual educational level seems to have little influence on the effect of area level deprivation. This indicates that individual SES and area level deprivation may act through different pathways [41]. Whereas individual SES may have a more direct influence on health (e.g. by providing individual resources for a healthier lifestyle), area level deprivation may act through a network of collective infrastructural resources such as potential access to places for physical exercise [2] or concepts such as the walkability of an area. Unequally distributed area-specific resources concerning the physical and built environment (e.g. the availability of green space, walking and cycling lanes), the social environment (e.g. availability of sport clubs, self-perceived neighbourhood safety) and health care (e.g. physician density) may contribute to inequalities in diabetes and obesity prevalence [42]. All these factors should be considered as they could contribute to an ‘obesogenic environment’ [43]. Disentangling area effects and identifying those with the strongest impact on health is still a major challenge, but it is crucial in order to find the starting points for future area-specific preventive measures.

However, the role of individual educational level should not be neglected. Having a low educational level may lead to low health literacy. This could result, for instance, in less benefit from diabetes disease management programmes [44] caused by a poorer understanding of health care instructions. It has often been demonstrated that the risk of T2DM is inversely related to educational level. This can only be partially explained by BMI, as shown, for example, in the EPIC-Interact study covering eight western European countries [3]. Also, it is well known that education influences obesity [45] and, following Sobal and Stunkard [46], that increasing SES is associated with decreasing obesity prevalence among women in developed societies. Thus, there is a need to focus preventive measures on both low SES population groups and highly deprived regions.

Our results show that the effects of individual SES and area level deprivation are more pronounced among women than among men. Kavanagh et al. [47] demonstrated that lower education and lower income were associated with higher biomarker levels of diabetes and cardiovascular disease as well as higher waist circumferences in women. Tang et al. [48] reported significant adjusted ORs for income and education in the association with self-reported diabetes for women. Roskam et al. [4] reported higher educational inequalities in obesity among women using health survey data from 19 European countries. Maier et al. [6] showed that the prevalence of T2DM in five German study regions was associated with area level deprivation at a municipality level in Germany, revealing slightly higher effects of individual SES and of area level deprivation among women than among men. Looking at the association between inner-city neighbourhood unemployment rates and the prevalence of T2DM in these five study regions, Müller et al. [13] also showed that the prevalence of T2DM was higher for women than for men with low SES but, in contrast, higher for men with a high neighbourhood unemployment rate compared with women. However, using the unemployment rate alone as an indicator for neighbourhood deprivation may lead to a potential gender bias.

Fano et al. [49] demonstrated that diabetes prevalence and area deprivation are directly related, particularly for women. However, sex differentials in diabetes diagnosis between men and women might be a possible explanation for stronger associations in women compared with men. Matheson et al. [50] have also shown that women living in the most deprived areas have a higher BMI than women living in the most affluent ones.

### Limitations and strengths

Some limitations of our study have to be taken into account. The potential for non-response bias has to be considered. Low educational level and diabetes have both been associated with non-response [51]. Study participation could also vary according to area level deprivation [52]. We assume that non-response could lead to underestimation of our results. All our variables, in particular diabetes and obesity prevalence, are self-reported and not validated, for example by medication or anthropometric measurements. This could lead to potential problems of misclassification [53]. There is evidence from previous studies that self-reported T2DM leads to very similar associations with SES as validated information on T2DM prevalence [54]. However, self-reported BMI tends to be underestimated, especially in the case of higher BMI values [55], which might have diluted the observed associations.

Education is well accepted as being a good indicator of individual SES. For example, a study conducted in nine European countries demonstrated that the relationship between overweight and low education is stronger and more consistent than for other SES variables such as household income [56]. However, in additional analyses (data not shown), we replaced education with household income. We found a very similar picture, for example a significantly increased OR for women in quintile 5 looking at the prevalence of T2DM (OR 1.21; 95% CI: 1.00–1.47).

Also, our analyses are based on districts. These administrative units vary considerably in area and population size. Therefore, the classification of individuals by area level deprivation may be more sensitive in smaller districts than in larger ones. Moreover, when assessing the association between area deprivation and health, the influence of the modifiable areal unit problem (MAUP) depends on the size of spatial units: using smaller areas (e.g. municipalities instead of districts) may provide even more significant results [57].

Finally, the cross-sectional design of the dataset does not allow any causal interpretation of our findings.

Some important strengths of our study should be pointed out. We used an extensive database, based on a large representative nationwide dataset including individual data from two consecutive national health interview surveys conducted across the whole of Germany. This is an excellent resource for studying regional differences in the prevalence of T2DM and obesity. Using an established area-based deprivation measure for Germany, we were able to quantify the effect of area deprivation on the prevalence of T2DM and obesity controlling for individual educational level. To our knowledge, this is the first study looking at the association of area level deprivation, T2DM and obesity covering the whole of Germany at a district level.

## Conclusion

In Germany, higher area level deprivation is associated with a higher prevalence of type 2 diabetes mellitus and obesity at the national level, independent of individual educational level and established risk factors. In order to reduce health disparities, diabetes and obesity prevention strategies need to consider individual as well as area-based risk factors [58]. For this, it will be necessary to identify the mechanisms underlying the individual and the area level deprivation components as well as their interactions.
